# Attitudes Toward Health, Healthcare, and eHealth of People With a Low Socioeconomic Status: A Community-Based Participatory Approach

**DOI:** 10.3389/fdgth.2021.690182

**Published:** 2021-07-08

**Authors:** Jasper S. Faber, Isra Al-Dhahir, Thomas Reijnders, Niels H. Chavannes, Andrea W. M. Evers, Jos J. Kraal, H. J. G. van den Berg-Emons, Valentijn T. Visch

**Affiliations:** ^1^Faculty of Industrial Design Engineering, Delft University of Technology, Delft, Netherlands; ^2^Faculty of Social and Behavioral Sciences, Leiden University, Leiden, Netherlands; ^3^Department of Public Health and Primary Care, Leiden University Medical Centre, Leiden, Netherlands; ^4^National eHealth Living Lab, Leiden University Medical Centre, Leiden, Netherlands; ^5^Erasmus School of Health Policy and Management, Erasmus University Rotterdam, Rotterdam, Netherlands; ^6^Department of Rehabilitation Medicine, Erasmus Medical Center (MC), University Medical Center Rotterdam, Rotterdam, Netherlands; ^7^Capri Cardiac Rehabilitation, Rotterdam, Netherlands

**Keywords:** low socioeconomic status, eHealth adoption, health attitudes, community-based participatory research, user profiles, health disparities, eHealth intervention design

## Abstract

Low socioeconomic status (SES) is associated with a higher prevalence of unhealthy lifestyles compared to a high SES. Health interventions that promote a healthy lifestyle, like eHealth solutions, face limited adoption in low SES groups. To improve the adoption of eHealth interventions, their alignment with the target group's attitudes is crucial. This study investigated the attitudes of people with a low SES toward health, healthcare, and eHealth. We adopted a mixed-method community-based participatory research approach with 23 members of a community center in a low SES neighborhood in the city of Rotterdam, the Netherlands. We conducted a first set of interviews and analyzed these using a grounded theory approach resulting in a group of themes. These basic themes' representative value was validated and refined by an online questionnaire involving a different sample of 43 participants from multiple community centers in the same neighborhood. We executed three focus groups to validate and contextualize the results. We identified two general attitudes based on nine profiles toward health, healthcare, and eHealth. The first general attitude, *optimistically engaged*, embodied approximately half our sample and involved *light-heartedness* toward health, *loyalty* toward healthcare, and *eagerness* to adopt eHealth. The second general attitude, *doubtfully disadvantaged*, represented roughly a quarter of our sample and was related to feeling *encumbered* toward health, feeling *disadvantaged* within healthcare, and *hesitance* toward eHealth adoption. The resulting attitudes strengthen the knowledge of the motivation and behavior of people with low SES regarding their health. Our results indicate that negative health attitudes are not as evident as often claimed. Nevertheless, intervention developers should still be mindful of differentiating life situations, motivations, healthcare needs, and eHealth expectations. Based on our findings, we recommend eHealth should fit into the person's daily life, ensure personal communication, be perceived usable and useful, adapt its communication to literacy level and life situation, allow for meaningful self-monitoring and embody self-efficacy enhancing strategies.

## Introduction

Low socioeconomic status (SES) is associated with a higher prevalence of unhealthy lifestyles compared to a high SES (1). Consequently, people with a low SES are at increased risk of chronic diseases (e.g., cardiovascular disease, diabetes, and obesity) (2–4). eHealth interventions such as monitoring devices, online communication platforms, and serious games have been proven effective in changing behavior and promoting a healthy lifestyle in various domains. However, these interventions are less successful in changing the behavior of people with a low SES due to low reach, less adherence during the intervention or less effectiveness of the interventions (5–9).

A crucial factor in facilitating the adoption, and therefore success, of eHealth interventions, is the alignment with a person's attitude toward using this technology (10, 11). Moreover, successfully achieving a lifestyle change, a primary goal of such interventions, requires the person to have a positive attitude toward their health and health services (12). eHealth is designed to expect its intended users to have a positive and pro-active health attitude. However, considering the growth of current health inequalities, such interventions would have a bigger impact when they can support groups not sharing these attitudes.

A multitude of studies point out that people with a low SES have unfavorable attitudes toward their health, healthcare, and eHealth. For instance, Wardle and Steptoe (13) found that health attitudes within the low SES groups are specifically characterized by a lower consciousness about health and less often thinking about the future. Other studies have identified more passive attitudes toward healthcare (14) and less confident attitudes toward digital health interventions (15) within low SES groups. Nevertheless, there is insufficient evidence to inform researchers and designers about these attitudes. The complexity of studying health values within contrasting sociodemographic environments poses various emotional and ethical challenges such as perceived harms, feelings of stigmatization, and anxiety toward research and the research team (16–18). As a result, hard-to-reach groups are minimally included in research efforts. Moreover, existing evidence is difficult to generalize toward other contexts. Measurements of attitudes are highly context-dependent and are expected to differ by country, setting, and time (19). Financial well-being and accessibility of health sources, for example, will not have a profound impact within countries that have unemployment funds, state-funded healthcare, and relatively good public transportation. Consequently, we have a lack of evidence to support the research and design of eHealth interventions that align with the attitudes of people with a low SES.

The rise of eHealth in current healthcare systems opens up exciting new possibilities to improve healthcare quality and efficiency. However, with the increased use of technical innovations and digital systems come unintended, unpredictable, and adverse consequences for individuals. Due to the underrepresentation of these specific societal groups, interventions are minimally aligned toward their attitudes. Consequently, these interventions face the risk of not being adopted and therefore unintentionally contribute to rising health inequalities. Researchers and designers should carry the responsibility to harness the potential of eHealth to create benefit for all groups in society, not merely for those that are motivated to perform a healthy lifestyle (20).

To engage the target group in the research process, an approach is needed that is comprehensive, culturally sensitive, and builds upon a relationship-based personal approach (18). Community-based participatory research (CBPR), a socio-culturally sensitive approach, which creates a trustful and long-lasting relationship between researcher and participant, has been effectively applied in culturally contrasting contexts (21, 22). For example, Henderson et al. (23) successfully implemented a CBPR approach to develop a tailored web-based diabetes self-management tool in a low-resource setting in the United States. Such an approach can engage hard-to-reach groups in the research process, yet has not been applied in the context of attitudes in low SES groups. In addition, focusing on a community instead of a person's individual characteristics is increasingly being recognized as a valuable approach. Studies that focus on these characteristics imply that these are the cause of poor health outcomes, which carries the risk of increasing stigma (24). It is becoming increasingly known that contextual community factors, such as the availability of healthy food, experiences of discrimination, and neighborhood poverty, also have a significant relation to poor health outcomes (25, 26).

The resulting knowledge could improve the alignment of health services toward attitudes of low SES populations, thereby facilitating their adoption. Currently, eHealth interventions aimed at these populations have only been minimally tailored, for example, by simplifying text and including images and videos (27). However, there is currently limited evidence reporting how interventions could be tailored toward psychological characteristics, such as attitudes with regard to eHealth. Although some studies report on the relationship between attitudes and interventions (28, 29), the resulting knowledge is difficult to apply in the design of interventions directly. Forms of practical knowledge, such as data-driven patient-profiles, have been used in the past to tailor content, context, and delivery of care toward individual preferences (30). Yet, such a form of knowledge has not been developed for attitudes of people with a low SES toward their health, healthcare, and eHealth in general.

This study aims to achieve design-relevant knowledge about the attitudes of people with a low SES toward their health, healthcare, and eHealth. To achieve this, we took a community-based participatory research approach to facilitate responsible engagement of the target group in the research process. The resulting knowledge can facilitate the design and alignment of health services toward the different attitudes of low SES populations. This will result narrowing current health disparities by developing interventions that are more acceptable, satisfactory, and user-friendly.

## Materials and Methods

**Figure 1:**
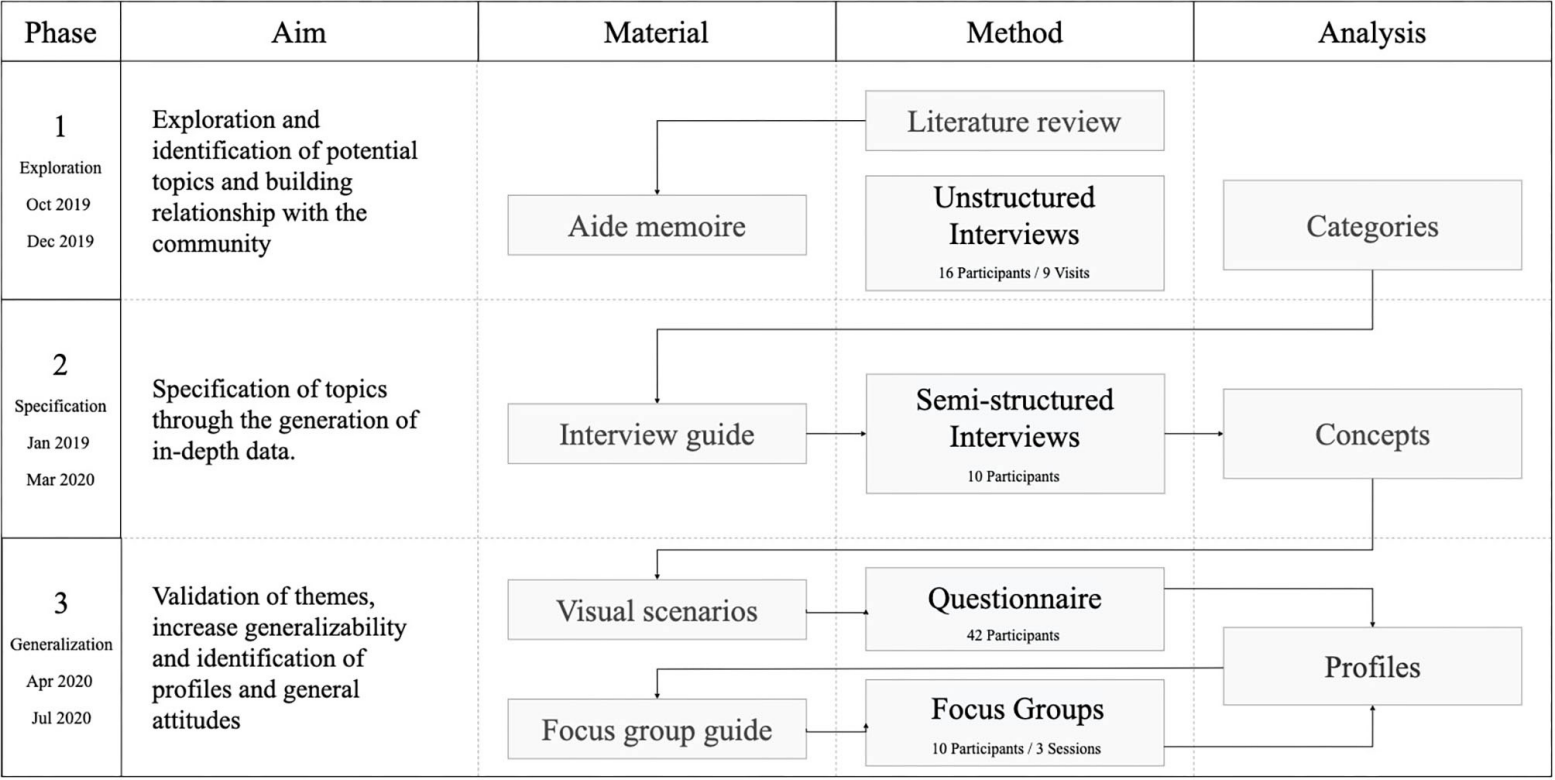
Overview of project phases and corresponding methods, materials, and analysis products.

Our methodology revolved around the principles of CBPR. CBPR is a partnership approach to research that equitably involves community members, organizational representatives, and researchers in all aspects of the research process (21). Our CBPR approach consisted of three separate phases (Figure 1) in which the outcomes of each phase were used in the next.

### Sampling and Recruitment

We initiated our collaboration with a community center located in a neighborhood in Rotterdam, the Netherlands. The neighborhood was selected based on its neighborhood SES, a combined measure of neighborhood income, education, and occupation (31). The neighborhood in which the community center is situated has been one of the lowest scoring neighborhoods on livability; a combined measure of its social, physical, and safety index (32). The area therefore is on the agenda as one of the *focus-neighborhoods* of the municipality of Rotterdam. Sixty-eight percent of the inhabitants have a migration background, compared to 52% in Rotterdam. In addition, 59% of the households have a low income compared to 52% in Rotterdam. Finally, 34% of the inhabitants have a low education, compared to 32% in Rotterdam (33).

The participants were sampled based on their affiliation with the community center and their living area (neighborhood SES). The community center situated in this neighborhood facilitates inhabitants that struggle with fundamental aspects of their life. They focus on poverty, occupation, living, social contacts, upbringing, and safety. We included participants living in the selected neighborhood with the following affiliations with the community center: (1) Visitors (Vi): Persons who visit the community center regularly and require support. (2) Volunteers (Vo): Unemployed persons who performed volunteering work in the community center in exchange for state funding. (3) Key persons (Kp): Social workers who have close relationships with the community members. In this study, Kp's were not considered as part of the target group as they are employed at the community center and are in the role of providing support. However, since they interact with Vi's and Vo's on a daily basis, we included them to learn about attitudes within the community from the Kp's perspective. In that light, we did not include Kp's in the second phase of the study as we were solely interested in acquiring a deeper understanding of the attitudes we observed in the first phase. Finally, it should be noted that Vo's could visit the community center as Vi's as well. For this study, we considered persons a Vo when they had at least one regular weekly shift at the community center.

In phase one, we sampled the participants conveniently and recruited them face-to-face at the community center. In the second phase, Vo's and Vi's were purposively sampled and recruited face-to-face. In phase three, we recruited participants for the questionnaire through an advertisement on the community center's Facebook page and WhatsApp group (Supplementary Figure 1) and through Kp's of various community centers within the same neighborhood. The participants for the focus groups were recruited through a question attached at the end of the digital questionnaire and by approaching them face-to-face at the community center. Because of the come-and-go nature of the community center, some participants frequently visiting the community center participated in each of the three phases, while others only participated in one.

### Ethics

The study protocol was approved by the Human Research Ethics Committee of Delft University of Technology (approval numbers 953, 1064, and 1141). Through our relationship-based CBPR approach we aimed to limit the impact of emotional and ethical challenges such as perceived harm, feelings of stigmatization, and anxiety toward research and the research team. In the first phase, we briefed our participants orally about the nature of the study as a formal written consent in this first introduction phase would obstruct a trustful interaction. The participants provided their consent verbally to the researcher (JF). In phases 2 and 3, when the relationship was more solid, written informed consent was provided.

### Procedure and Materials

In phase one, we aimed to form a trustful research partnership with the community and narrow down the research scope by simultaneously exploring and identifying specific research directions. We initiated the partnership by attending community gatherings and organizing health-themed lunch events at the community center. Such immersive activities have been used and proven successful in creating a relationship in various other CBPR efforts (21). During these activities, we addressed the research scope by engaging in unstructured interviews with community members individually. Based on an initial literature review, a backlist of topics guided the interviews and helped to steer them toward our research questions (34). We divided the topic questions into three overarching research themes: attitudes toward health, healthcare, and eHealth. For example, we explored the attitude toward health with questions such as “*How important is it for you to live long?”*. Questions such as “*What do you think of your doctor's advice?”* and “*What do you think of a technology that could help you live healthier?”* referred to the attitude toward healthcare and eHealth, respectively. The full interview backlist is provided in Supplementary Table 1. Data was captured by taking quick field notes during the visits and elaborating on them into comprehensive reports directly afterward.

In phase two, we investigated the specific directions resulting from the first phase more extensively through semi-structured interviews. In contrast to unstructured interviews, these interviews are more formal and intimate, which comes conjointly with emotional challenges when discussing sensitive and stigma-inducing topics (35). Therefore, the pre-established trusting relationship between participants and the researcher was an essential facilitator. The interviews (*N* = 10) were conducted at the community center in a separate room with the participants individually and took ~30 min. We developed the interview guide structuring the interview based on the research directions from the first phase. For example, we explored how the participants perceived their health with the question: “*What do you have to do to become 100% healthy?”*. The interview guide is provided in Supplementary Table 2. The data was collected by audio-recording and transcribing the interviews. We progressed to the subsequent phase when we achieved theoretical saturation.

**Figure 2:**
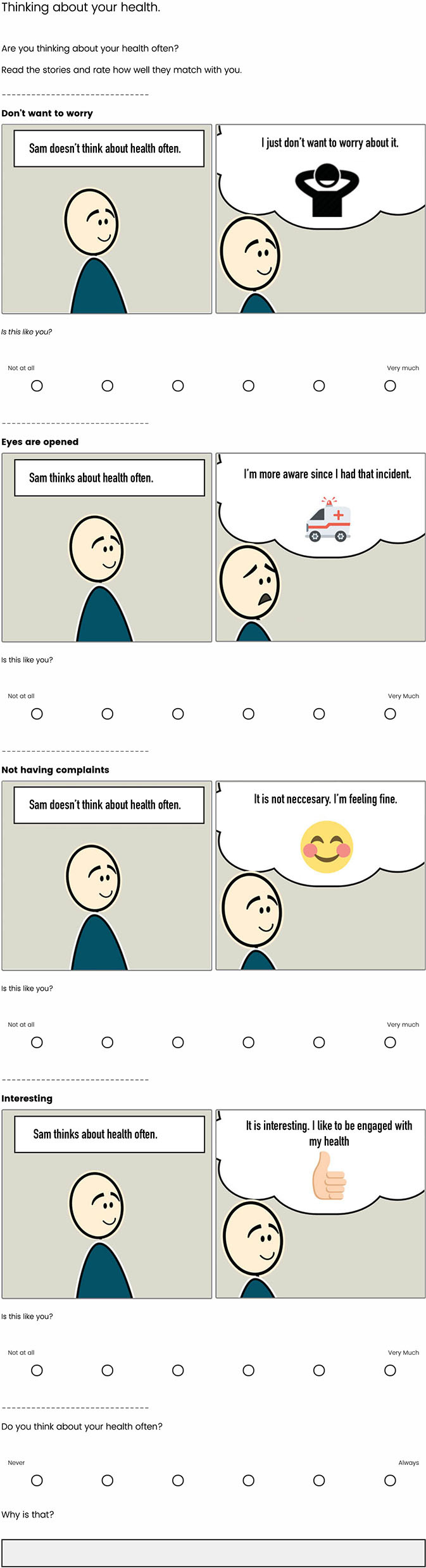
An example of the visual questionnaire distributed in phase 3. The storyboards represent the concepts found within the consciousness category.

In the third phase, we validated and generalized the insights from phase two and discovered general attitudes through the data-driven profiles. Meanwhile, we had to consider the newly introduced COVID-19 regulations. Therefore, we developed a digital questionnaire which we distributed digitally to members of community centers. This questionnaire presented the resulting insights of the second phase and asked the participants to rate the extent to which they felt the insight reflected themselves. By distributing this online questionnaire, we reached a more extensive and diverse sample. In addition, we gathered quantitative data that we used to validate our preliminary results and develop data-driven attitude profiles. Questionnaires, frequently being long and textual, are at risk of being disengaged by their participants as they depend on reading comprehension. This risk holds especially true for participants with lower education attainment. The use of graphics in previous studies has successfully engaged low-literate participants with questionnaires (36). Therefore, we synthesized our insights toward visual two-frame storyboards. We executed several pilot sessions to reduce the chance that participant understandings would not match the story's original implication and adjusted any inaccuracies accordingly. A 6-point Likert scale accompanied the stories in the questionnaire. The stories were grouped under their representative category. Each group concluded with an open-ended question regarding the corresponding category. See Figure 2 for an example of the *consciousness* page in the questionnaire. In addition, we asked participants to report their age, gender, educational attainment, and neighborhood. The online questionnaire was designed and distributed using Qualtrics. Finally, we performed focus groups to validate and contextualize the profiles that resulted from the questionnaire. Each focus group meeting consisted of three to four participants, lasted for ~1 h, and was audio-recorded. The focus groups took place in a large and ventilated room at the community center that allowed maintaining 1.5-m distance between the participants according to the COVID-19 regulations.

### Data Analysis

In phases one and two, we transcribed the audio recordings verbatim and analyzed them together with the field reports and qualitative questionnaire data using the software package Atlas Ti. Throughout the qualitative analysis, we followed the grounded-theory approach outlined by Corbin and Strauss (37), as it is specifically useful in discovering social processes focused on social change and improvement (38). We continuously broke down the data and collected it under similar content in the form of *concepts* using open coding techniques. For example, we created the concept *perceived barriers* to refer to quotes where participants mentioned barriers that decreased their motivation to perform healthy behavior. Subsequently, we grouped related concepts toward overarching *categories* based on attitude theory constructs such as Beliefs, Feelings, Motivation, and Opportunity (19, 39). Two independent researchers (JF and IA) developed the concepts together to improve the reliability of the results.

In phase three, we imported the Likert scores of the concepts and categories obtained from the questionnaire as variables into SPSS. We performed k-means cluster analyses on the concepts based on Euclidian distance for health, healthcare, and eHealth with SPSS. We determined the optimal number of clusters with the Elbow method using the factoextra and NbClust packages in R. We used an ANOVA to identify the concepts with significant (*p* < 0.05) contribution to the cluster segmentation. The concepts with an insignificant contribution were removed from further analysis. To validate the clusters, we performed an ANOVA with the category scores as independent and the clusters themselves as dependent variables. Using a *post-hoc* ANOVA, we defined the resulting clusters based on significant differences between mean scores of the concept variables. We created profiles by further clarifying and enriching these clusters by analyzing the qualitative data from the questionnaire and focus group discussions. This was done by extending on the existing categories and concepts and using the same grounded-theory approach as used in previous phases. Supplementary Table 4 shows an overview of the coding used for characterizing the profiles. Finally, we performed a principal component analysis (PCA) using the factoextra package in R to discover correlations between concepts from different profiles.

## Results

### Participants

During the unstructured interviews in the first phase, we spoke with 16 different members of the community center. These members consisted of eight Vi's, two Vo's and six Kp's. In the second phase, we interviewed five Vo's and five Vi's. In phase one and two, we did not collect demographic data. In the third phase, 45 participants responded to the questionnaire. From these latter responses, we excluded three participants not living in our target neighborhood from analysis. The participants' mean age in this final sample was 52 years (SD = 11.10), 21% was male and 79% was female. Most of this sample (67%) had a low to medium education, which was defined as not having attained a follow-up education. This is relatively high compared to 59% in the Netherlands. Ten participants participated in the focus groups; two Kp's, five Vo's, and three Vi's.

### Phase 1 and 2: Exploration and Specification

The unstructured interviews of phase one yielded 30 pages of field reports containing 85 coded segments. The semi-structured interviews of phase two yielded 10 interview transcripts containing 359 coded segments. The grounded theory analysis resulted in 58 concepts within nine categories related to attitudes toward health, healthcare, and eHealth. Examples of the categories found are: *consciousness* about health, *motivation* to perform healthy behavior and *satisfaction* toward healthcare. Examples of identified concepts are: *Interest* in health, *Perceived barriers*, and *loyalty* toward healthcare provider. Table 1 presents an overview of the concepts and categories included in the third phase. We excluded categories conveying a limited number of concepts (*N* = 1) or not fitting the attitude theory constructs (*N* = 1). We selected the concepts to include (*N* = 29) in the third phase based on the number of associated coded segments and discussion by the two analysts.

**Table 1:** Concepts (*N* = 29) under their categories (*N* = 9) resulting from grounded theory analysis including number (*N*) of associated codes, description, and exemplary quotes (translated).

**Concept**	***N***	**Description**	**Quote**
**Category: health beliefs [being healthy is…]**			
Working on health	30	When one frequently performs healthy behavior such as physical activity and maintaining a healthy diet.	“*I'm eating healthy, I only drink in the weekends […] I frequently do yoga […] Yes I think that I'm being healthy” (Vo3)*
Absence of complaints	12	The absence of complaints, symptoms, and disease.	“*There was a time when I was heavier. I struggled with shortness of breath and cholesterol and I don't know what else.” (Vi6)*
Participation	12	Being able to go out and participate in society.	“*The first thing you have to do is to get up early and just go somewhere […] Otherwise you will not have active contacts with people who provide a positive influence or create chances for you” (Vi3)*
Balance	10	Maintaining a balance between unhealthy and healthy behavior.	“*I have other things. I don't drink for instance so that makes up for it quite a lot.” (Vo5)*
Life under control	10	When you have a roof above your head and no major financial or social struggles.	“*Unhealthy is when you don't have a roof above your head and you have to roam the streets.” (Vi5)*
**Category: consciousness [about health is impacted by…]**			
Complaints	19	The experience of health-related symptoms and complaints.	“*I haven't visited the doctor in 30 years. My last painkiller I used when I was at high school” (Vo5)*
Incident	13	The consideration of a health-related incident in the past.	“*Yes, a significant impression. Before that [the incident] I was just flying blind.” (Vo3)*
Concern	11	The extent to which one is concerned about their health.	“*You can come up with all sorts of graphs, but I don't, I just don't want to worry about it. Maybe it is just very easy the way I live.” (Vi5)*
Interest	3	The level of interest one has in their health.	“It doesn't interest me […]. I just eat whatever I like” (Vi3)
**Category: motivation [to perform healthy behavior is impacted by…]**			
Future perspective	22	The consideration of its value toward future health.	“*How important is the future for you?” “Well, I just hope to continue like this.” (Vo3)*
Perceived barriers	20	The amount of financial, social, and environmental barriers one perceives.	“*I have always had a one-sided diet. A lot of cheese for example. We didn't have a fridge at work.” (Vi1)*
Feeling	6	The extent to it contributes to the subjective emotional state one experiences.	“*Do you think it's important to do it [performing healthy behavior]?” “Yes, it makes you feel better.” (Vo2)*
Enjoyment	5	The extent to which it impacts the level of joy in one's life.	“*No, I don't really consider it [being healthy] that much. You also would want to enjoy life” (Vo4)*
**Category: control [one perceives to have over health is impacted by…]**			
Support	24	The amount of support one receives on managing their health.	“What facilitates you in doing it [healthy behavior]?” “To be honest, my friend. […] She supports me and shows me the ropes.” (Vo2)
Self-efficacy	14	The level of capabilities one perceives to have to change health-related behavior	“*But you are not eager to quit, are you?” “I am my boy, however, I'm not able to. If you have a pill for me that I take and it makes me quit…” (Vi5)*
Chance	13	The belief that what happens regarding health is all based on chance and coincidence.	“*I'll not reach the age of 110, I'm not that healthy. Although, it doesn't say much actually because there are people who are 100 years old and they still smoke.” (Vi5)*
Fatalism	5	The belief that what happens regarding one's health is subjugated to fate or destiny.	“*You can't really do something about it [getting sick]. The only thing you can do is watch out [for accidents], that is the only thing.” (Vi3)*
**Category: healthcare experience [is impacted by…]**			
Communication	13	The quality of communication with the healthcare provider.	“*I would like them to take more time for people like me, who do not fully understand it. Sometimes I really feel like a foreigner.” (Vo2)*
Autonomy	11	The need one has to deserve autonomy within the healthcare process.	“*I proposed it [not eating meat] once, however, my general practitioner told me not to do it. […] He didn't go into depth or asked me why I wanted that. He just advised me to keep eating meat” (Vo4)*
Authority	10	The amount of authority one perceives their healthcare provider to have.	“*It is not possible to change something about it yourself. If they say there is no solution then it has to be that way […] yes you know, they are the doctor.” (Vo1)*
Personal	4	The need one has to be treated on a personal and humane level.	*It was a nice guy, a physiotherapist. He always brought a ball of Feyenoord (football club in the Netherlands). […] Yes, the soccer I liked. However, all the other stuff, walking around, walking with a sack, and all of that. At a certain moment, I thought like. man. (Vi5)*
**Category: messages [reception is impacted by…]**			
Source Interpretation	4	The extent to which one perceives a conflict between different sources (e.g., healthcare, media, social environment) of health messages.	“*The website that you visit… It can be that someone wrote it at home and it is not true. However, it can also be a doctor who wrote it, in that case, it is true.” (Vo1)*
Nuances	2	The extent to which one can understand and apply nuances within health messages.	*Yes, sugar, I have to minimize. […] Everything I have to minimize. Also, Carbohydrates. (Vi1)*
Rules	2	The extent to which one interprets health messages as rules.	“*Recently we have had this [healthy-lunch café] […] Everything must be healthy, and you are not allowed to eat meat. Well, I really like my piece of meat […]. In that case, just let me be unhealthy. I don't care; I just really like it.” (Vi5)*
Doctor as information source	2	The fact that the health-related information came from a healthcare professional or not.	“I won't go and try out stuff from the internet and stuff. It has to come from the doctor.” (Vo1)
**Category: eHealth [intention to use is impacted by…]**			
Enthusiasm	7	The belief in the positive aspects and potential of eHealth	“*Yes, I find that really interesting. […] You just have to ask google what you have to do. For example, I bumped my toe a few times, and then you get an answer.” (Vi5)*
Anxiety	7	The level of anxiety one experiences toward (prospective) usage of eHealth.	“*It is too complicated. […] They told me I had to download something. Well, they did it for me. I don't know how it works.” (Vo2)*
Exposure	5	The extent to which a person is exposed toward eHealth.	“*I'm not entirely up to date what it can mean to me. Maybe I'm still thinking in the old way. I don't know what I'm missing.” (Vi3)*
Trust	1	The level of trust in technology and its related privacy and safety risks.	“*I always try to protect myself with anti-virus software […] If you have your gates open, you will collect all kinds of unwanted rubbish” (Vi2)*

### Phase 3—Generalization

#### Profiles

**Figure 3:**
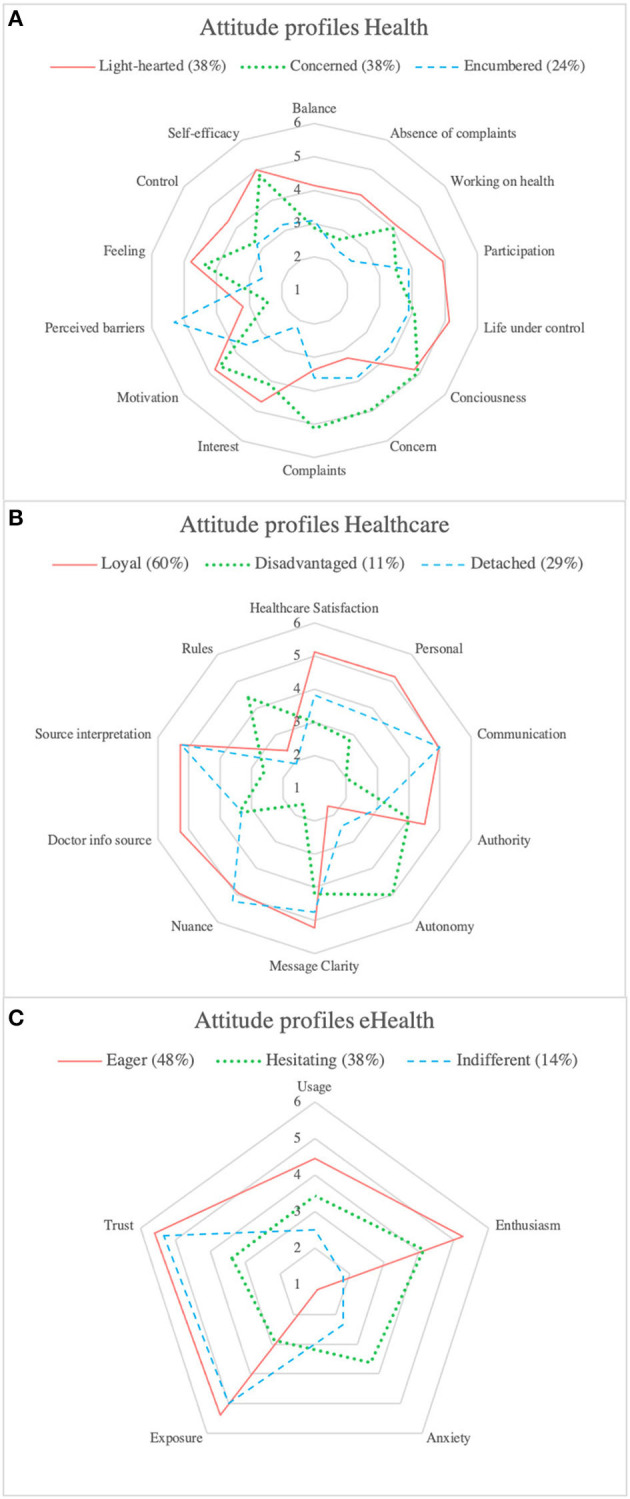
**(A)** Radar graph of concept means of the attitude profiles toward health. **(B)** Radar graph of concept means of the attitude profiles toward healthcare. **(C)** Radar graph of concept means of the attitude profiles toward eHealth.

The descriptive analysis of the overall sample revealed a high variance in the means of the different concepts. Variance ranged from 0.80 to 3.78 with a mean of 1.91. Therefore, it was all the more essential to investigate a segmented version of the data. The elbow method suggested that three clusters best segmented the data of each topic. We found significant differences between the category means, indicating the validity of our clusters. Figure 3 presents a graphical representation of the mean scores characterizing the clusters and Supplementary Table 3 offers a detailed overview. Table 2 shows the demographic information of overall sample and the profiles. Although we found significant differences between the means of the concepts, we did not find significant differences between the clusters' demographic variables.

**Table 2:** Questionnaire respondent characteristics.

	***N***	**CV**	**Age**		**Gender %**		**Education %**	
			**M**	**SD**	**Male**	**Female**	**Low**	**High**
Sample	42	1.91	52	11.10	21	79	67	33
Concerned	16	1.46	54	9.70	31	69	75	25
Light-hearted	16	1.06	48	13.07	0	100	69	31
Encumbered	10	2.00	51	8.89	40	60	50	50
Loyal	25	1.06	53	10.81	24	76	68	32
Detached	12	1.05	48	8.62	0	100	58	42
Disadvantaged	5	2.01	48	12.54	60	40	80	20
Eager	20	1.21	48	12.44	15	85	65	35
Hesitant	16	1.72	55	8.34	37	63	75	25
Indifferent	6	1.96	52	12.10	0	100	50	50

Regarding attitudes toward health, the majority was represented by the *Light-hearted* and *Concerned* profiles (both 38%), which were characterized by higher scores on *consciousness, motivation*, and *feeling*. The Concerned profile was differentiated based on higher scores for *concern, complaints*, and lower *control*. The *Encumbered* profile represented lower scores on *consciousness, motivation, self-efficacy*, and *interest* and higher scores on *perceived barriers*.

For the attitudes toward healthcare, the *Loyal* profile (60%) was the most significant. This profile was marked by higher scores on *satisfaction, personal, authority*, and *doctor as information source*. The *Disadvantaged* profile was characterized by lower scores on *satisfaction, communication, source interpretation, nuance, personal* and higher scores on *rules* and *autonomy*. The *Detached* profile contained no specific concept that differentiated it from the other profiles.

Regarding attitudes toward eHealth, the *Eager* (48%) and *Hesitating* (38%) profiles represented the majority of the attitudes and were both characterized by a higher score on *enthusiasm*. The *Hesitating* profile could be differentiated based on lower scores on *usage, trust*, and *exposure* and a higher score on *anxiety*. The *Indifferent* profile was marked by lower scores on *usage* and *enthusiasm*.

#### Qualitative Enrichment

The qualitative data from the questionnaire responses and three focus group discussions clarified and enriched the profiles with contextual information. Table 3 presents representative quotes for each profile. Regarding the health profiles, within the *Concerned* profile, 81% of the questionnaire participants referred to the experience of medical complaints, symptoms, and limitations as a reason for being more conscious about health. Within the *Light-hearted* profile, 69% of the questionnaire participants referred being healthy and seeing the importance of it. What stood out within the *Encumbered* profile was that 50% of the questionnaire participants expressed not enjoying healthy behavior and experiencing internal barriers regarding motivation. During the focus group discussions, we found that most participants recognized themselves with the *Light-hearted* and *Concerned* profiles. It stood out that some participants mentioned recognizing periods of the *Concerned* profile, especially when experiencing medical complaints or limitations. The participants did not fully identify with the *Encumbered* profile but rather ascribed this to an attitude they had in the past, frequently seen in the youth, or an attitude they “sometimes” have.

“*Sometimes I have, just like [Encumbered], my concerns about things. In that case you can find yourself in a slump. Life is not always going your way.” (Vi10)*

**Table 3:** Exemplary quotes per profile.

**Profile**	**Quote**
Light-hearted	“*I do what I can and what I want. When I feel good, it is good.”*
Concerned	“*I try to prevent my health complaints from taking over my life. It is tough sometimes though […]”*
Encumbered	“*Exercising is exhausting and painful”*
Loyal	“*I feel that they listen well to me. Everything is explained clearly. Messages are clear and informative.”*
Disadvantaged	“*They left me for too long with my complaints, and I'm not taken seriously”/“Sometimes they come with difficult words”*
Detached	“*I'm not coming to the doctor often, but when I do, I have the feeling they listen well. Probably extra because I never visit the doctor.”*
Eager	“*I see it as a push in the back, and it's fun to keep track of things. I'm already above 950 km this year:”*
Hesitating	“*The technology of nowadays is more something for the younger generation”*
Indifferent	“*Not feeling like it”*

Regarding the profiles toward healthcare, within the *Loyal* profile, 92% of the questionnaire participants referred to positive experiences such as good advice, a professional who shows understanding, and additional room for questions and discussion. Within the *Detached* profile, 46% of the questionnaire participants mentioned distrusting their doctors and not visiting them often. For the *Disadvantaged* profile, 67% of the questionnaire participants referred to communication barriers such as lack of time, complicated language, feelings of anxiety, and not being taken seriously. During the focus groups, the participants could identify with the *Loyal* and *Detached* profile. Regarding the Detached profile, which we positioned as an attitude not wanting to be dependent on healthcare, we gathered additional evidence that some of our participants were distrusting and wanting to avoid healthcare:

“*Yes, I think I am a bit like [Detached]. Because I am not a doctor visitor. I seldom visit the doctor. […] I do not really like to take medication. Only when it is really necessary.” (Vi11)*

Regarding the profiles toward eHealth, within the *Eager* profile, 75% of the participants referred to using eHealth and seeing the benefits of using it. Although we also found such positive responses toward eHealth within the *Hesitating* profile (56%), 38% of this profile's participants also referred to eHealth as not worth the effort, better suited for the youth, or being perceived more like gadgets. The *Indifferent* profile hosted participants referring to not wanting to be involved with technology for health (50%). During the focus groups, most participants identified with the *Eager* and *Hesitating* profiles. What stood out was that some participants who initially were *Indifferent* toward eHealth started to become interested in it because of the focus group discussion:

“*Well, I definitely want to use it. Suppose I can do it with a device or something. My daughter also wanted to install an app for counting steps. However, I don't do a lot with phones. It is only now that we have this conversation that I start to think that maybe I should investigate it some more. I only use it for calling and text messaging. I do like it, but I don't know it.”* (Vi11).

#### General Attitudes

**Figure 4:**
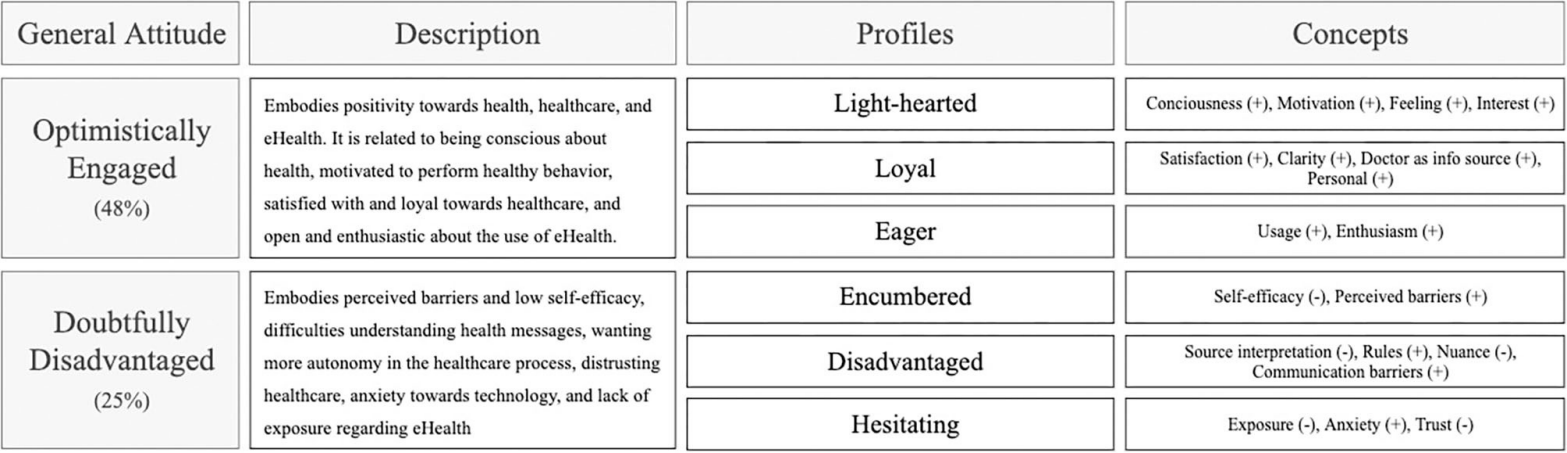
Overview of the general attitudes resulting from the principal component analysis and their corresponding profiles and concepts.

By investigating the inter-profile relationships, we could identify two attitudes toward health, healthcare, and eHealth in general. Figure 4 displays an overview of these attitudes. Correlation coefficients can be found in Supplementary Table 5. The most significant general attitude, *Optimistically Engaged* could be described by positivity toward health, healthcare, and eHealth. It is related to being conscious about health, motivated to perform healthy behavior, satisfied with and loyal toward healthcare services, and open and enthusiastic about the use of eHealth. It was defined by the relationship between the characterizing scores of the *Light-hearted (consciousness, motivation, feeling*, and *interest), Loyal (satisfaction, clarity, doctor as info source, and personal)*, and *Eager (usage* and *enthusiasm)* profile. The average size, based on the questionnaire respondents, of the combination of these profiles is 48%. The second general attitude, *Doubtfully Disadvantaged*, reflected perceived barriers and low self-efficacy, difficulties understanding health messages, wanting more autonomy in the healthcare process, distrusting healthcare, anxiety toward technology, and lack of exposure regarding eHealth. It was defined by the relationship between the scores of the *Encumbered (low self-efficacy* and *perceived barriers), Disadvantaged (source interpretation, rules, nuance*, and *communication barriers)*, and *Hesitating (exposure, anxiety*, and *trust)* profile. The average size, based on the questionnaire respondents, of the combination of these profiles is 25%. The *Concerned, Detached*, and *Indifferent* profiles did not have any specific relations with other profiles. They should be seen as individual profiles that could exist in any combination with other profiles. However, the *concerned* profile's substantial representation within the questionnaire respondents (38%) makes it important to consider further. This profile was characterized by the experience of complaints, high concern, and low feelings of control because of the experience of a health-related incident or continuous experience of health complaints.

## Discussion

### Main Findings

This study aimed to develop design-relevant knowledge about the attitudes of people with a low SES toward their health, healthcare and eHealth. Through a CBPR approach consisting of three phases, we identified two general attitudes based on nine distinct profiles. This knowledge could be used to develop a better understanding of existing attitudinal knowledge and to propose design recommendations that facilitate the alignment of health services toward these attitudes.

### Relevance and Implications

Since most of the attitudes toward health, healthcare, and eHealth were positive, we believe that there is a willingness from a large part of the target group to adopt eHealth interventions to improve their lifestyle. Nevertheless, we discovered a diverse range of different attitudes that have different implications for the design of eHealth interventions. The attitudes represented by the profiles can be used to develop design recommendations to improve the alignment of eHealth interventions toward attitudes of low SES groups.

#### Optimistically Engaged

The profiles (*Light-hearted, Loyal, and Eager*) represented by this general attitude have similarities and contradictions with existing literature. The *Light-hearted* profile was represented by high consciousness about health. Contrastingly, other studies found that low SES populations have a less conscious attitude toward health and think less about the future (13, 40). Complex social situations, caring responsibilities (29), and time and energy constraints (28) result in little room to act toward and think about long-term investments such as a healthy lifestyle. These contrasting findings could be explained by the current living situation of our participants. Almost all participants were either retired, unemployed or disabled and therefore were not constrained by their jobs or worried about finances as they receive financial support from the government. In Wardle and Steptoe (13), all participants were employed, and in Coupe et al. (29), only 13% of the population was employed. Yet, the finding came from interviews with healthcare providers and not from the low SES population themselves. In a previous study in a community center in Rotterdam, participants indicated that a lack of time was a major reason for not visiting a community center (41). Therefore, we argue that some participants in our sample, having the time to visit a community center, also had more time and capacity to think about and act toward a healthy lifestyle. Therefore, we recommend that eHealth researchers and designers should become aware of the person's life situation and use this knowledge to determine whether the person has the capacity available to fit the intervention into their life. People that do not have this capacity would benefit more from services that deliver support in social or financial aspects (42, 43). We argue that people that do have motivation and consciousness could benefit from being empowered to play a major role in their health management. This could be achieved through shared-decision making, providing health information and facilitating self-management (44). It remains important for healthcare providers to be aware of this attitude as it is known that clinician perceptions of patients with a low SES have been shown to affect clinical decision making. Based on common beliefs about people with a low SES, physicians tend to delay diagnostic testing, prescribe more generic medications and avoid referral to specialty care and potentially lifestyle interventions (45). The finding that most of our participants were doctor dependent (*Loyal*, 60%) conforms to other studies that claim that people with a low SES are loyal to and rely on their doctor's advice (14, 46). Moreover, we found that our participants highly valued a personal interaction with their care provider. The importance of this personal touch is mentioned in various other studies on the interaction between people with low SES and healthcare providers (43, 47–49). Since current healthcare systems are moving from a doctor-says, patient-does model toward a model of shared decision making and self-management, we expect that people relying on their doctor's advice will experience increasing difficulties in their health management. To improve the alignment of eHealth communication to these attitudes, we recommend that professionals should be mindful of “dehumanizing” healthcare, as digital interactions lack the nuances of human interaction (50). Therefore, eHealth interventions should be designed to incorporate and enhance personal communication, interaction, and relationships with care providers, family members, and peers. This could be done for example by integrating a social role in the intervention through interactive and animated computer characters. Through simple speech, hand gestures and other non-verbal cues, these characters could simulate face-to-face counseling to establish trust and rapport in a virtual environment (51).

#### Doubtfully Disadvantaged

The *Encumbered, Disadvantaged*, and *Hesitating* profile, that represented this attitude, all embodied a perceived lack of control related to one's health, healthcare, or eHealth. Various previous studies support this finding. The lack of control over health is attributed to lower problem-solving skills (52), environmental deprivation (53), and financial, environmental, and social limitations (40, 54). Therefore, we recommend considering self-efficacy and perceived control enhancing strategies within eHealth interventions. Goal setting has been mentioned as a potentially successful strategy in various studies regarding other low SES populations (8, 29, 47). A possible implementation is through persuasive game design. Through the game world the user could acquire feelings of competence and transfer these toward the real world (55). For example, one could help an avatar to progress through different life goals by earning points based on healthy snack choices (56). In addition, various studies also mention social support as a potentially effective strategy (28, 43). Emotional support could be offered through supportive conversations and buddy systems, informational support from educational information from peers and providers and appraisal could be offered through peers, providers, or the eHealth system itself (57). In addition, designers could think of ways to make technologies and information more accessible and easier to integrate into the persons' daily life. For example, cardiac telerehabilitation allows to reach patients in their home-environment and motivate them to participate even though they do not have the means (physically as well cognitive) to visit the rehabilitation center (58, 59). We found that participants characterized by the *Disadvantaged* profile were experiencing communication difficulties in the healthcare setting. Especially assessing and applying health knowledge was perceived as problematic. It is striking that this profile only represented a mere 12% of our sample, while these difficulties are widely discussed in previous studies on this topic (46, 60). Since our participants were proficient in the Dutch language, we argue that communication for them was less problematic. Moreover, combatting health literacy is currently high on the agenda (61). In fact, in the Netherlands, 60% of healthcare professionals report adapting their communication toward their patients' needs (62). Nevertheless, to include this part of the population, eHealth interventions should accommodate for varying literacy levels, for example by using visual aids and plain language. Besides, according to studies related to other low-SES and literacy populations, medical advice should be tailored to increase its relevance (28, 48, 49). For example, by using lab results to select the appropriate advice given in a patient portal (48).

The participants within the *Hesitating* profile reported being unsure about using eHealth because they were unaware of how it could be of personal value. A previous study found that people who have a poor understanding of what eHealth can do for them have little interest in signing up and using it (50). It also seems that healthcare providers do not actively promote such interventions and provide little encouragement to use them, as they expect the intervention will not be adopted (29). In addition, this subgroup of participants expressed concerns about not being capable enough to use eHealth. This finding is also reflected by Latulipe et al. (48), where most usage concerns of low-income older adults relate to the difficulty of initially logging on to a system. Therefore, we recommend professionals to consider the perceived usefulness and usability of the eHealth intervention. Past studies have shown that this can be achieved through supportive healthcare providers and peers who can promote the eHealth interventions and provide technical assistance during usage (48, 50, 63). One upcoming medium through which these interactions can take place is through social media. Social media is used as an effective recruitment and engagement medium for eHealth applications (50) and for people with lower income and education (64). Another possibility to improve perceived usability is by offering primary task support through self-monitoring wearable devices (e.g., activity trackers) (65), reduction (e.g., list with food choices), or tunneling (e.g., offer treatment opportunities after an interactive test about tobacco addiction) (66).

#### The Concerned Profile

The participants represented by the *Concerned* profile indicated being motivated and conscious because they were living with medical limitations or have recently experienced a health-related incident. This concerned attitude could serve as potential entry point for researchers and designers to motivate healthy behavior. While people might already be aware of the susceptibility and severity of getting a disease, they might benefit from convenient cues to action such as reminders and suggestions provided either by a peer, professional, or system (67). According to Bukman et al. (28), people with a low SES are especially motivated by the feedback they receive from their bodies. This conforms to some participants mentioning that their attitude had changed throughout their lives, resulting from experiencing health complaints or incidents. Therefore, it is challenging to motivate these individuals to engage in preventive behavior when they do not yet perceive complaints. Therefore, following Bukman et al. (28), we recommend that for people that do not have the concern (yet), feedback should be provided in a visual, meaningful, and directly applicable way that conforms to the beliefs of the target group. According to Orji et al. (67), self-monitoring, simulation and personalization and tailoring strategies are effective to help individuals develop accurate perceptions of own risk. Nevertheless, we could argue that data recorded by most activity trackers and self-monitoring applications currently is still of little value in facilitating meaningful reflection on lifestyle. In a previous study it was found that the participants from a low SES neighborhood rarely analyzed their self-monitoring experiences to derive insight about the meaning of data for their well-being (68). One example of providing meaningful data is a smoking app that displays, besides the number of days without cigarettes, also the amount of money the person has saved by not smoking.

### Recommendations

Based on our results, the reflection with previous literature, and existing recommendations, we propose some final recommendations for improved eHealth alignment to attitudes in low SES populations. First, we have identified a large part of our sample embodied an optimistic and engaged general health attitude. According to this attitude, someone is motivated, conscious, satisfied with healthcare, and open toward eHealth. Hence, we expect that for this attitude, healthcare services, and interventions are generally appropriate.

However, we also identified attitudes that are less in line with our current processes and expectations. We identified profiles that embodied a disinterested, resisting attitude toward healthcare (*Detached*) and eHealth (*Indifferent*). We argue that tailoring eHealth interventions toward such attitudes is resource-intensive and would be more effective when directed at attitudes that are positive yet require support. These attitudes, in our study identified under the *doubtfully disadvantaged* general attitude, currently seem to hold the most potential for tailoring efforts. While the Encumbered profile benefits from social and emotional support, the disadvantaged profile benefits from additional support in understanding verbal and written health information and guidance during the healthcare process. The Hesitating profile has an open yet unsure attitude toward eHealth and therefore benefit from supportive and technology promoting healthcare professionals and peers. We recommend professionals to focus on these attitudes specifically, to become aware of the corresponding needs, and subsequently use and design eHealth as a tool to respond to these needs. While doing so, professionals are advised to establish a trustful relationship with the target group, which could be achieved through personal contact and/or through trusted doctors or other key persons (18). In addition, future research endeavors should take into account the challenges related to recruiting and researching vulnerable populations and take the appropriate methodological strategies to minimize the impact of those challenges. This could help improve the accessibility and affordability of eHealth innovations and thereby help equalizing inequalities in healthcare.

### Strengths and Limitations

This study addressed the ever-increasing gap in health disparities by giving voice to a target group that is frequently overlooked in health research. Traditional approaches have received criticism as they, when executed irresponsibly, bring forth mistrust, feelings of stigmatization, and anxiety (69). CBPR has gained increasing attention in addressing ethical challenges in health research, as it encourages equity and shared decision-making and increases community involvement (21). By taking this approach, we ensured that our participants felt comfortable, safe, and especially involved during the research activities. The resulting insights directly carry our participants' voices and are, therefore, a meaningful contribution to responsible digital health. While frequently people with a low SES are expected to adapt their attitudes toward the intervention, we aim to have a more complete idea of how we should design interventions to be adapted to them.

Although our study provides an in-depth insight into the attitudes of people living in a low SES neighborhood, the results are not generalizable toward all low SES contexts. First, we aimed at limiting possible feelings of stigmatization by sampling on neighborhood SES. This would make it difficult to relate the findings directly to other studies that select participants on individual measures of SES (i.e., education, income, and occupation). Yet, this different selection criterion allowed us to target a group that would otherwise have been excluded. For example, the questionnaire demographics indicate a relatively high percentage of participants who attained a follow-up education. In traditional studies, this part of the sample would have been seen as high-SES and therefore excluded from the study. Socioeconomic determinants and barriers leading to disparities in health behavior are complex (54, 70). Capturing them merely based on individual determinants is therefore problematic and has accumulated critique over the years (71). Instead, our focus on neighborhood SES takes into account other factors that have proven to have a significant relation to poor health outcomes (i.e., availability of healthy food, experiences of discrimination, and neighborhood poverty) (25, 26).

Another factor that could impact the generalizability is the context of the community center. According to an earlier report of another community center near Rotterdam, 36% of the visitors were unemployed (72). This percentage is significantly higher than the neighborhood in general (9.4%) and Rotterdam (7%) (73). Since our participants had the motivation to visit the community center, they could also have been more motivated to perform healthy behavior. Finally, it should be taken into account that this study has taken place in an urban context with sufficient governmental support, developed infrastructure, and social support. Therefore, the results are not directly applicable to countries that do not have these facilities. While the results themselves might not be directly generalizable to other low SES populations, they provide a deep and contextualized understanding of a sample of the target group that can be applied in the design of eHealth interventions. According to Crouch and McKenzie (74), such research inquiries in naturalistic settings often seek to discover social insights that extend beyond initial observations. This requires the researcher to be immersed in the research field, establish continuing fruitful relationships with respondents and through theoretical contemplation to address the research problem in depth. A small number of cases will facilitate the researcher's close association with the respondent. A review of CBPR approaches in the health domain confirms this statement as it reports sample sizes of roughly the same order of magnitude (75). Future research could be aimed at generalizing the results (e.g., profile characteristics) in larger-scale sample sizes. Finally, the concepts identified in this research are, although informed by supporting themes in literature, data driven and not a priori based on a specific theory or model. Hence, they provide a deeper layer and a supplementary perspective to existing knowledge. Nevertheless, researchers should act with discretion when interpreting the resulting insights using existing theory.

### Conclusion

To develop successful eHealth interventions that support people with a low SES in achieving a healthy lifestyle, it is crucial to consider their attitude toward this technology and their health and healthcare in general. This study explored attitudes of people living in a low SES neighborhood toward their health, healthcare, and eHealth using a community-based participatory research approach. This unique approach helped us discover novel and bottom-up insights that strengthen our current understanding of these attitudes. This understanding allows researchers and designers to have a more nuanced view of the attitudes in low SES populations. Intervention developers should be mindful of differentiating life situations, motivations, healthcare needs, and eHealth expectations. eHealth should fit into the person's daily life, ensure personal communication, be perceived as usable and useful, adapts its communication to literacy level and life situation, allow for meaningful self-monitoring and embody self-efficacy enhancing strategies. When these recommendations are taken into account when developing eHealth interventions for people with a low SES, these interventions' alignment with their attitudes will improve. This will result in interventions that are more acceptable, satisfactory, and user-friendly. Consequently, eHealth interventions will finally move from widening toward narrowing current health disparities and thus align with societal health responsibilities.

## Data Availability Statement

The raw data supporting the conclusions of this article will be made available by the authors, without undue reservation.

## Ethics Statement

The studies involving human participants were reviewed and approved by the Human Research Ethics Committee (HREC)-TU Delft. The participants provided their written informed consent to participate in this study.

## Author Contributions

JF, IA-D, TR, AE, NC, HB, JK, and VV contributed to conception and design of the study. JF planned and executed the CBPR process. IA-D and JF were involved in the analysis and interpretation of the data. JF was responsible for the writing of the manuscript. All authors contributed to manuscript revision and read and approved the submitted version.

## Conflict of Interest

The authors declare that the research was conducted in the absence of any commercial or financial relationships that could be construed as a potential conflict of interest.
