# Supplementary material for: Attitudes Toward Health, Healthcare, and eHealth of People With a Low Socioeconomic Status: A Community-Based Participatory Approach
Source: Front Digit Health. 2021 Jul 8;3:690182. doi: 10.3389/fdgth.2021.690182 (PMC8521920; doi:10.3389/fdgth.2021.690182)
Supplement: Supplementary file 1 [file Data_Sheet_1.docx]

Supplementary Material

## Supplementary Figures


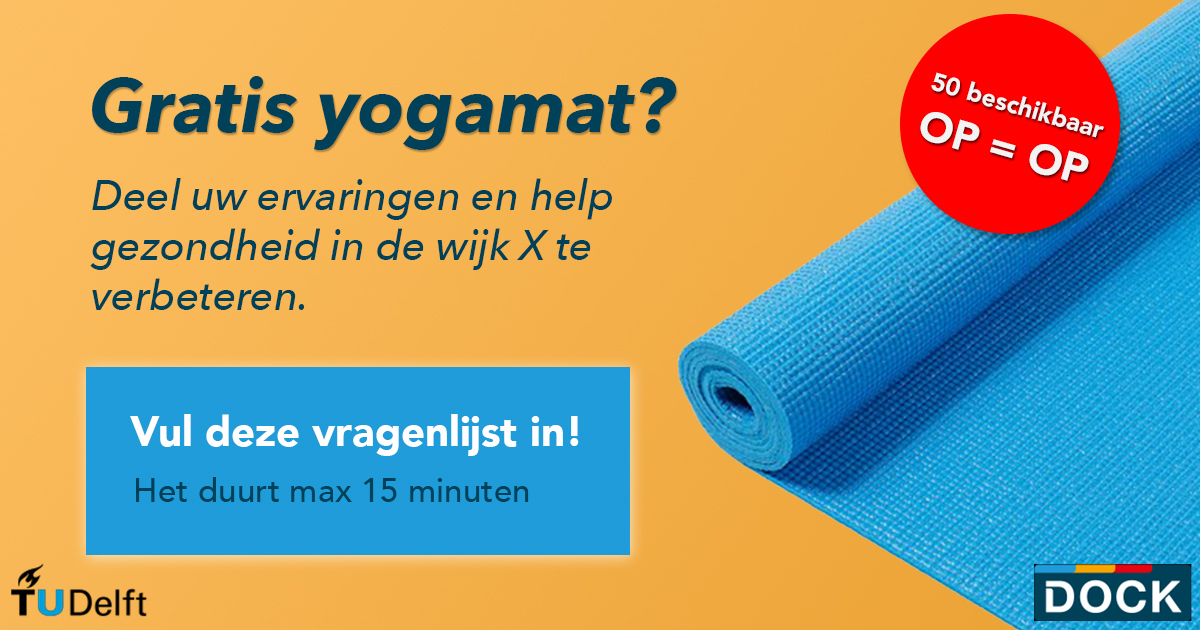


**Supplementary Figure 1.** Recruitment message. Translated: Free yoga mat? Share your experiences and help to improve health in the X neighborhood. Fill in this questionnaire. It will take a maximum of 15 minutes.

## Supplementary Tables

| **Themes** | **Question** |
| --- | --- |
|  |  |
| 1.1 Start-up | How are you doing? |
| 1.2 Personal information | What is your age |
|  | What kind of work do you do? |
|  | What kind of education have you followed? |
|  | Are you born in the Netherlands? |
| 1.3 Living situation | Could you tell me something about your living situation? |
|  | Where do you live? How does your home look like? With whom do you live? |
| 1.4 Grand Tour | Can you take me through a typical day? |
|  |  |
| 2. Health | **Connection topics: Eating, Smoking, Alcohol, Sports, Illness, Social wellbeing, stress, incidents from the past.** |
| 2.1 Start-up | Do you feel healthy? |
|  | Why is that? |
|  | Would you like to change something? |
|  | At what point did you consider yourself healthy? |
| 2.2 Lifestyle | Do you play sports? Or some other kind of activity? |
|  | What do you take into account when you are eating? What is healthy? What is unhealthy? |
| 2.3 Perception | What is according to you a healthy life? |
|  | What are, according to you, the three most important aspects of your health? |
|  | What does your healthy looks like next year? |
| 2.4 Attitude | Do you think about your health often? |
|  | Are you ever tempted to choose for enjoyment instead of long-term health? |
| 2.5 Future | Do you think it’s important to be healthy when you are old? |
|  | What do you want to invest? |
|  | Why is it so important for you? |
| 2.6 Past | Did you ever experience something serious regarding your health? |
|  | Who or what supports you in maintaining your current healthy? |
|  | When you look back, would you have done it differently? |
|  | Do you ever face regret from decisions you have made in the past? |
|  | Are there things you feel you didn’t have control over? |
| 2.7 Barriers | Can you name any barriers in your environment? |
|  | Are there persons that work against you? |
| 2.8 Facilitators | Are there things that help you in your environment? |
|  | Are there persons that help you? |
| 2.9 Knowledge/Skill | What would you like to know more about being healthy? |
|  | Who or what do you go to if you want to know more about your health? |
|  | Can you name things you would like to do but can’t manage? |
|  |  |
| 3. Healthcare | **Connection points: Medication, Surgery, Doctor, Hospital, General Practitioner** |
| 3.1 Start-up | Do you have experience with coming to the doctors or hospitals? |
| 3.2 Healthcare | Can you name an example? |
|  | What did you like about this experience? |
|  | How could this experience have been improved? |
| 3.3 Care provider | What kind of advices do you receive from the doctor? Do you agree with them? Do you adhere to them? |
|  | Is the doctor always right? |
|  | Did you ever have a discussion with your doctor? |
|  | Do you feel free to engage in such a discussion? |
|  | What do you think the doctor thinks of you? Does he/she take you seriously? |
|  | Is the doctor putting effort into making sure you understand everything? |
|  | Would you like to see your doctor more often? Have more time with him/her? |
| 3.4 Health messages | Who do you listen to? Who not? |
|  | What do you think of all the attention revolving around being healthy nowadays? |
|  |  |
| 4. eHealth | **Connection points: Technology, Smartphone, apps, tv, digital, online banking, internet, websites.** |
| 4.1 Technology in general | What technologies do you use on a daily basis? |
|  | Why do you use them? |
|  | What do you like about them? |
|  | Are you often frustrated? |
|  | How could your experience be improved? |
|  | If you think of technological innovations, what appeals to you? |
|  | Why are you not yet using it? |
|  | What do you definitely not want to use? |
| 4.2 Technologies for health | Do you ever use a technology for your health? What do you think of it? |
|  | How could you use technology to live healthier? |
|  | Would you trust the technology? And when your doctor recommends it? |
|  | What could be improved according to you? |
| 4.3 Characteristics of technology | What is according to you important when using a technology. |
|  | How should it look like? What does it consist of? |

**Supplementary Table 1.** Unstructured interview backlist (Translated into English)

| **Question** | **Category** |
| --- | --- |
| Do you think you are healthy at this moment? | Intro/Ice-breaker |
| Are you engaged with your health?  You think health is interesting? | Consciousness |
| When would you call yourself 100% healthy?  Would you like to be 100% healthy?  What would you have to do to achieve this?  What could be reasons for not wanting this? | Health Beliefs  Motivation  Motivation  Motivation |
| When would you call yourself unhealthy?  What do you have to do to prevent this from happening?  Why is it important for you to not become unhealthy?  What helps you to achieve this?  How important is it for you to be healthy even when you are old?  If you knew what you know now, would have lived in a different way in the past? | Health Beliefs  Motivation  Motivation  Control  Motivation  Consciousness |
| What are the chances that you will become seriously ill?  And in 5 years?  Do you think this is important?  What can you do about this? | Control    Motivation  Control |
| Have you ever been in touch with healthcare?  What did you think of this experience?  What should be improved according to you? | Healthcare Experience |
| Do you talk with others about your health?  What advices do you listen to? | Messages |
| What technologies do you use on a regular basis?  What do you think of these technologies?  What could be improved?  Do you use technology specifically for your health?  What do you like about them? Why not?  Would you trust such technologies? Even when the doctor recommends them? | eHealth |

**Supplementary Table 2.** Semi-structured interview guide (Translated into English)

| **Health** | | | | | | | | |
| --- | --- | --- | --- | --- | --- | --- | --- | --- |
| **Questionnaire** | | | | | **Focus Groups** | | | |
| P | **Beliefs** | **Consciousness** | **Motivation** | **Control** | **P** | **Code** | | |
| **Concerned** | | | | | | | | |
| 1 | Absence of complaints, Life under control, Mental wellbeing, Participation | Complaints | Perceived Barriers | Health complaints impact control | Vo7 | Complaints | | |
| 2 | Mental wellbeing | Automatic | Feeling | Lack of internal control | Vo1 | Complaints | | |
| 3 | Mental wellbeing, Participation | Complaints | Ability | Health complaints impact control | Vo3 | Incident, periodically, staying healthy, future perspective, health complaints impact control | | |
| 4 | Working on health | Complaints | Future perspective | Health complaints impact control | Vi10 | Periodically | | |
| 5 | Direct Feedback Working on health | Complaints | Direct Feedback | N/A |  |  | | |
| 6 | Life under control, Mental wellbeing, Working on Health | Complaints | Perceived Barriers | Own responsibility Chance |  |  | | |
| 7 | Absence of complaints  Mental wellbeing | Future Perspective | Family | Own responsibility |  |  | | |
| 8 | Absence of complaints | Complaints | Security | N/A |  |  | | |
| 9 | Working on health | Complaints | N/A | N/A |  |  | | |
| 10 | Feeling, Mental wellbeing, Working on health | Complaints | Future perspective | Health complaints impact control Own responsibility |  |  | | |
| 11 | Absence of complaints, Ability | Fatalism | Body benefit | Chance, Fatalism |  |  | | |
| 12 | Absence of complaints | Complaints | Family | Health complaints impact control |  |  | | |
| 13 | N/A | Complaints | N/A | Chance |  |  | | |
| 14 | Absence of complaints | Complaints | Feeling | Motivation is control |  |  | | |
| 15 | Mental wellbeing, Working on health | Important | N/A | Own responsibility |  |  | | |
| 16 | Important | Complaints | Perceived Barriers | Health complaints impact control |  |  | | |
| **Light-hearted** | | | | | | | | |
| 17 | Mental wellbeing Working on health | Important | Health doesn't come for free | Motivation is control Important | Vo7 | Important, Carefree | | |
| 18 | Important | Future Perspective | Age Medical Requirement | Own responsibility Chance | Vo1 | Carefree | | |
| 19 | Absence of complaints | Future Fear | Age | Own responsibility Important | Vo8 | Carefree, No complaints | | |
| 20 | Absence of complaints Mental wellbeing | Staying Healthy Feeling | Working on health | Own responsibility | Vi10 | No complaints | | |
| 21 | Mental wellbeing | Complaints | Enjoyment | Support | Vi11 | Feeling | | |
| 22 | Important | Ability | Staying healthy | Own responsibility Working on health |  |  | | |
| 23 | Participation Working on health | Complaints | N/A | Health complaints impact control |  |  | | |
| 24 | Future Perspective | Complaints | Family | Own responsibility |  |  | | |
| 25 | Enjoyment | Lack of internal control | Balance | Health complaints impact control Own responsibility |  |  | | |
| 26 | Mental wellbeing Working on health | Future Fear | Security | Own responsibility |  |  | | |
| 27 | Absence of complaints Life under control Mental wellbeing Working on health | Family Body benefit Important | Family | Own responsibility |  |  | | |
| 28 | Life under control Mental wellbeing Working on Health | Life under control | Perceived Barriers | Life under control |  |  | | |
| 29 | Ability Abscence of complaints Mental wellbeing Participation | Fatalism Future perspective | N/A | Own responsibility |  |  | | |
| 30 | Mental wellbeing Working on health | Important | Future Perspective Staying healthy | Own responsibility |  |  | | |
| 31 | Working on health Participation | Future Fear | Habit | Chance |  |  | | |
| 32 | Ability Feeling | Feeling Relaxed | Feeling | Feeling |  |  | | |
| **Encumbered** | | | | | | | | |
| 33 | Perceived barriers | Complaints | Future Perspective | Own responsibility | Vo5 | lack of Internal control self-efficacy | | |
| 34 | Ability | Complaints | Lack of internal control | Own responsibility Chance | Vi10 | lack of Internal control Youth | | |
| 35 | Absence of complaints Working on health Participation | N/A | Lack of internal control | Health complaints impact control | Vi11 | Perceived barriers | | |
| 36 | Mental wellbeing Working on health | Complaints | N/A | Health complaints impact control | Vo7 | Youth No interest | | |
| 37 | Absence of complaints | Complaints | Medical requirement | Medical requirement | Kp7 | Youth Perceived barriers | | |
| 38 | Absence of complaints | N/A | Perceived Barriers | Lack of internal control | Vo8 | Not taking responsibility lack of Internal control | | |
| 39 | Ability | Lack of internal control | Perceived Barriers | Chance | Kp7 | self-efficacy | | |
| 40 | Working on health | Future fear | N/A | Lack of internal control | Vo3 | Perceived barriers | | |
| 41 | Absence of complaints | Engagement | Lack of internal control | Lack of internal control |  |  | | |
| 42 | Important | Complaints | Perceived Barriers | Health complaints impact control |  |  | | |
|  | | | | | | | | |
| **Healthcare** | | | | | **eHealth** | | | |
| **Questionnaire** | | | **Focus Groups** | | **Questionnaire** | | **Focus Groups** | |
| **P** | **Healthcare** | **Messages** | **P** | **Codes** | **P** | **Codes** | **P** | **Codes** |
| **Detached** | | | | | **Eager** | | | |
| 14 | Lacking Service | Additional Information | Vi11 | Not visiting doctor, not wanting medication, autonomy | 16 | Using eHealth | Vo1 | Monitoring, Gadget, not using ehealth |
| 15 | Authority Listening | Lacking Service | Vi10 | Negative experience, Unpersonal | 28 | Enthusiasm | Vi6 | Monitoring, eHealth motivation |
| 16 | Lacking Service | Lacking Service | Vo5 | Not wanting medication, Distrust, autonomy | 29 | Using eHealth | Vo5 | eHealth motivation |
| 26 | Authority | Additional Discussion Listening | Vi6 | Distrust, Unpersonal | 30 | Usability | Vo8 | Monitoring |
| 27 | Satisfied | Additional Discussion Clear Explanation | Vo7 | Unpersonal | 31 | eHealth motivation |  |  |
| 28 | Clear explanation Personal Taking time | N/A |  |  | 32 | eHealth motivation |  |  |
| 29 | N/A | N/A |  |  | 6 | Seeing Value of eHealth |  |  |
| 30 | Additional Discussion | Additional Discussion |  |  | 7 | eHealth motivation |  |  |
| 31 | Not visiting often | Clear explanation |  |  | 8 | Monitoring |  |  |
| 32 | Not visiting often | Clear explanation |  |  | 9 | Monitoring |  |  |
| 41 | Personal | Communication |  |  | 10 | Monitoring |  |  |
| 42 | Personal | Personal |  |  | 11 | Monitoring |  |  |
| **Disadvantaged** | | | | | 22 | Not motivated to use eHealth |  |  |
| 12 | Autonomy | Communication | Kp8 | Communication | 23 | Not motivated to use eHealth |  |  |
| 13 | No time | Communication | Vo1 | Communication | 24 | Monitoring |  |  |
| 38 | Not visiting often | N/A | Vo3 | Communication | 25 | Monitoring Using eHealth |  |  |
| 39 | N/A | Advice not effective |  |  | 34 | Not motivated to use eHealth |  |  |
| 40 | Communication | Communication |  |  | 35 | Using eHealth |  |  |
| **Loyal** | | | | | 36 | Using eHealth |  |  |
| 1 | Powerlessness | Clear explanation | Vo7 | Personal, Clear explanation | 37 | Not using |  |  |
| 2 | Authority Personal | Scientific Backup | Vo1 | Clear explanation | **Hesitating** | | | |
| 3 | Listening | N/A | Vi10 | Clear explanation, Listening, Personal | 14 | Using eHealth | Vo7 | Gadget, seeing value, technology anxiety |
| 4 | Personal | N/A | Vi6 | Personal | 26 | eHealth motivation | Vo1 | Gadget, not using, technology anxiety |
| 5 | Clear explanation | Clear explanation | Vi11 | Personal | 41 | Not seeing the value | Vi10 | Seeing value |
| 6 | Additional Discussion | Clear explanation |  |  | 12 | Enthusiasm | Vi11 | Seeing value, technology anxiety |
| 7 | Personal | Satisfied |  |  | 13 | N/A | Vi6 | Technology anxiety |
| 8 | Additional Discussion | Clear explanation |  |  | 38 | Not motivated to use eHealth Not seeing the value |  |  |
| 9 | Clear explanation | Additional Discussion |  |  | 39 | Monitoring |  |  |
| 10 | Personal | N/A |  |  | 1 | N/A |  |  |
| 11 | Powerlessness | Additional information Clear explanation |  |  | 2 | Monitoring No long-term motivation |  |  |
| 17 | Authority | See improvement |  |  | 3 | Monitoring |  |  |
| 18 | Additional Discussion Satisfied | Listening |  |  | 4 | Monitoring |  |  |
| 19 | Clear explanation Listening | Clear explanation |  |  | 17 | Not seeing value |  |  |
| 20 | Listening | Additional Discussion Clear Explanation |  |  | 18 | Not using |  |  |
| 21 | Additional Discussion Listening | Clear explanation |  |  | 19 | Enthusiasm |  |  |
| 22 | Listening | Clear explanation |  |  | 20 | Monitoring |  |  |
| 23 | Clear explanation | Listening |  |  | 33 | Not motivated to use eHealth |  |  |
| 24 | Clear explanation | Personal |  |  | **Indifferent** | | | |
| 25 | Listening | Clear explanation |  |  | 15 | Not seeing value | Vi11 | Not motivated to use eHealth |
| 33 | Additional Discussion Clear Explanation | Scientific Backup |  |  | 27 | Using eHealth | Vi10 | Not seeing value, not motivated to use ehealth |
| 34 | Taking time | Clear explanation |  |  | 42 | Using eHealth | Vo1 | Not motivated to use eHealth, Not seeing value |
| 35 | Personal | N/A |  |  | 40 | Technology anxiety |  |  |
| 36 | Listening | Additional Discussion Clear Explanation |  |  | 5 | Positive Feedback |  |  |
| 37 | Clear explanation | Clear explanation |  |  | 21 | Not motivated to use eHealth |  |  |

**Supplementary Table 3.** Qualitative codes resulting from the questionnaire and focus groups used for profile clarification and enrichment in phase 3.

| **Personal Health** | **1) Light-hearted**  **38%** | | **2) Concerned**  **38%** | | **3) Encumbered**  **24%** | |  |
| --- | --- | --- | --- | --- | --- | --- | --- |
|  |  |  |  |  |  |  |  |
|  | **M** | **SD** | **M** | **SD** | **M** | **SD** | ***P < 0.05*** |
|  | | | | | | | |
| **Category: Perception** | | | | | | | |
| Balance | 4.13 | 1.03 | 2.88 | 1.09 | 3.10 | 1.60 | 1 – (2,3) |
| Absence of complaints | 4.19 | 1.52 | 2.69 | 1.20 | 2.40 | 1.71 | 1 – (2,3) |
| Working on health | 4.13 | 0.81 | 4.00 | 1.16 | 2.40 | 0.97 | 3 – (1,2) |
| Participation | 4.94 | 0.68 | 3.50 | 0.89 | 3.90 | 1.29 | 1 – (2,3) |
| Life under control | 5.13 | 0.72 | 4.06 | 1.06 | 3.90 | 1.66 | 1 – (2,3) |
|  | | | | | | | |
| **Category: Consciousness** | | | | | | | |
| Consciousness | 4.81 | 0.54 | 4.94 | 1.00 | 3.80 | 1.32 | 3 – (1,2) |
| Concern | 3.25 | 1.00 | 4.94 | 1.06 | 3.90 | 1.52 | 2 – (1,3) |
| Complaints | 3.63 | 1.09 | 1.88 | 0.96 | 3.40 | 1.43 | 2 – (1,3) |
| Interest | 4.69 | 0.87 | 4.13 | 1.03 | 2.20 | 1.03 | 3 – (1,2) |
|  | | | | | | | |
| **Category: Motivation** | | | | | | | |
| Motivation | 4.81 | 0.54 | 4.63 | 1.09 | 3.60 | 1.17 | 3 – (1,2) |
| Perceived barriers | 3.19 | 1.17 | 2.44 | 1.26 | 5.30 | 1.06 | 3 – (1,2) |
| Feeling | 4.81 | 0.83 | 4.38 | 0.62 | 2.60 | 1.17 | 3 – (1,2) |
|  | | | | | | | |
| **Category: Control** | | | | | | | |
| Control | 4.31 | 1.20 | 3.31 | 1.35 | 3.20 | 1.40 | 1 – (2,3) |
| Self-efficacy | 5.00 | 0.73 | 4.81 | 1.28 | 3.20 | 1.69 | 3 – (1,2) |
|  |  |  |  |  |  |  |  |
|  | **4) Loyal**  **60%** | | **5) Disadvantaged**  **11%** | | **6) Detached**  **29%** | |  |
| **Healthcare** |  |  |  |  |  |  |  |
|  |  |  |  |  |  |  |  |
| **Category: Healthcare** | | | | | | | |
| Healthcare satisfaction | 5.12 | 0.83 | 3.00 | 1.41 | 3.83 | 1.03 | 4 – (5,6) |
| Personal | 5.16 | 0.69 | 2.80 | 1.10 | 3.70 | 0.99 | 4 – (5,6) |
| Communication | 4.96 | 1.42 | 2.00 | 1.00 | 5.00 | 0.85 | 5 – (4,6) |
| Authority | 4.52 | 1.33 | 4.00 | 1.58 | 3.00 | 0.95 | 4 – (5,6) |
| Autonomy | 1.68 | 0.80 | 5.00 | 0.71 | 2.42 | 1.24 | All |
|  | | | | | | | |
| **Category: Messages** | | | | | | | |
| Message clarity | 5.24 | 0.60 | 4.20 | 1.10 | 4.75 | 1.14 | 4 - 5 |
| Nuance | 4.92 | 1.38 | 1.60 | 0.55 | 5.25 | 0.87 | 5 - (4,6) |
| Doctor info source | 5.28 | 0.74 | 3.40 | 1.67 | 3.33 | 1.16 | 4 – (5,6) |
| Source interpretation | 5.28 | 0.98 | 2.60 | 2.07 | 5.25 | 1.06 | 5 - (4,6) |
| Rules | 2.40 | 1.12 | 4.40 | 2.07 | 1.92 | 0.90 | 5 - (4,6) |
|  |  |  |  |  |  |  |  |
|  | **7) Eager**  **48%** | | **8) Hesitating**  **38%** | | **9) Indifferent**  **14%** | |  |
| **eHealth** |  |  |  |  |  |  |  |
|  |  |  |  |  |  |  |  |
| Usage | 4.45 | 1.87 | 3.44 | 1.63 | 2.50 | 2.34 | 7 - 9 |
| Enthusiasm | 5.25 | 0.85 | 4.13 | 1.08 | 1.83 | 0.75 | All |
| Anxiety | 1.15 | 0.37 | 3.62 | 1.08 | 2.33 | 1.50 | All |
| Exposure | 5.40 | 1.14 | 2.87 | 1.36 | 5.00 | 0.90 | 8 – (7,9) |
| Trust | 5.60 | 0.50 | 3.37 | 1.31 | 5.33 | 0.82 | 8 – (7,9) |

**Supplementary Table 4.** Mean scores of and significant relationships (P < 0.05) between the nine profiles based on questionnaire concepts.

|  | **Satisfaction** | **Clarity** | **Doc. Inf.** | **Personal** | **Usage** | **Enthusiasm** |
| --- | --- | --- | --- | --- | --- | --- |
|  |  |  |  |  |  |  |
| **Consciousness** | -0.10 | 0.25 | 0.07 | -0.07 | **0.47** | **0.25** |
| **Motivation** | **-0.28** | 0.90 | -0.20 | -0.01 | **0.25** | **0.28** |
| **Feeling** | **0.26** | 0.00 | 0.14 | **0.31** | **0.28** | **0.44** |
| **Interest** | **0.30** | **0.28** | **0.31** | **0.26** | **0.38** | **0.30** |
|  |  |  |  |  |  |  |
|  | **Self-efficacy** | **Source Int.** | **Rules** | **Nuance** | **Comm.** |  |
|  |  |  |  |  |  |  |
| **Exposure** | **0.50** | **-0.58** | **-0.56** | **-0.48** | 0.25 |  |
| **Anxiety** | **-0.39** | **0.69** | **0.39** | **0.36** | **-0.50** |  |
| **Trust** | **0.33** | **-0.43** | **-0.48** | -0.21 | 0.06 |  |

**Supplementary Table 5.** Correlation coefficients between concepts. Significant correlations (P < 0.05) are highlighted.
